# Experimental observation of Earth’s rotation with quantum entanglement

**DOI:** 10.1126/sciadv.ado0215

**Published:** 2024-06-14

**Authors:** Raffaele Silvestri, Haocun Yu, Teodor Strömberg, Christopher Hilweg, Robert W. Peterson, Philip Walther

**Affiliations:** ^1^ University of Vienna, Faculty of Physics, Vienna Center for Quantum Science and Technology (VCQ), Vienna, Austria.; ^2^ University of Vienna, Faculty of Physics and Vienna Doctoral School in Physics, Boltzmanngasse 5, A-1090 Vienna, Austria.; ^3^ University of Vienna, Faculty of Physics and Research Network Quantum Aspects of Space Time (TURIS), Boltzmanngasse 5, A-1090 Vienna, Austria.; ^4^ Institute for Quantum Optics and Quantum Information (IQOQI) Vienna, Austrian Academy of Sciences, Boltzmanngasse 3, A-1090 Vienna, Austria.

## Abstract

Precision interferometry with quantum states has emerged as an essential tool for experimentally answering fundamental questions in physics. Optical quantum interferometers are of particular interest because of mature methods for generating and manipulating quantum states of light. Their increased sensitivity promises to enable tests of quantum phenomena, such as entanglement, in regimes where tiny gravitational effects come into play. However, this requires long and decoherence-free processing of quantum entanglement, which, for large interferometric areas, remains unexplored territory. Here, we present a table-top experiment using maximally path-entangled quantum states of light in a large-scale interferometer sensitive enough to measure the rotation rate of Earth. The achieved sensitivity of 5 μrad s^−1^ constitutes the highest rotation resolution ever reached with optical quantum interferometers. Further improvements to our methodology will enable measurements of general-relativistic effects on entangled photons, allowing the exploration of the interplay between quantum mechanics and general relativity, along with tests for fundamental physics.

## Teaser

Two maximally quantum entangled photons can sense that the Earth is rotating.

## INTRODUCTION

For more than a century, interferometers have been important instruments to experimentally test fundamental physical questions. They disproved the luminiferous ether, helped establish special relativity (*1*, *2*), and enabled the measurement of tiny ripples in space-time itself known as gravitational waves (*3*). With recent advances in technology, interferometers can nowadays also operate using various different quantum systems including electrons (*4*, *5*), neutrons (*6*), atoms (*7*–*11*), superfluids (*12*, *13*), and Bose-Einstein condensates (*14*–*16*). Quantum interferometers are of interest for two main reasons: First, the exploitation of quantum entanglement allows for super-resolving phase measurements that go beyond the standard quantum limit (*17*, *18*). Second, the enhanced sensitivity of quantum interferometers opens up opportunities for precision measurements that can explore new frontiers in physics. These include setting constraints on dark-energy models (*19*), testing quantum phenomena in non-inertial reference frames (*20*–*22*), and investigating the interplay between quantum mechanics and general relativity (*23*–*28*).

Optical systems are particularly well suited for realizing quantum interferometers, owing to mature techniques available for generating a variety of quantum states, ranging from squeezed vacuum (*29*–*32*) to maximally path-entangled photons (*33*–*35*). The N00N states belong to the latter category, represented by $\frac{1}{\sqrt{N}}\left({|N\rangle }_{a}{|0\rangle }_{b}+{|0\rangle }_{a}{|N\rangle }_{b}\right)$ , wherein *N* photons exist in a superposition of *N* photons in mode *a* with zero particles in mode *b*, and vice versa (*17*). These states behave similar to those of a single photon with *N* times the energy, enabling precision in phase measurements at the Heisenberg limit that scales as 1/*N* and thus goes beyond the $1/\sqrt{N}$ scaling of the standard quantum limit (*18*). Another advantage of photonic systems is that fiber-optical interferometers offer a clear pathway for expanding the interferometric area while maintaining a low level of quantum decoherence.

In this work, we report the design and operation of a large-scale quantum-optical fiber interferometer exploiting N00N states that reaches a sensitivity in the range of μrad s^−1^, sensitive enough to measure the rotation of Earth. We inject two-photon N00N states into a 715-m^2^ Sagnac interferometer, using quantum interference to demonstrate super-resolution while extracting Earth’s rotation rate. This goes beyond previous laboratory demonstrations of measurements probing Sagnac interferometers with quantum states of light, which involved fiber interferometers with at most hundred-meter-length fibers (*20*–*22*, *32*, *36*) and were only used to measure synthetic and controllable signals. We are able to confirm an acquired Sagnac phase from Earth’s rotation with an enhancement factor of two because of the two-photon entangled state. To the best of our knowledge, this is the largest and most sensitive quantum-optical Sagnac interferometer in the world, surpassing previous state-of-the-art rotation sensors using two-particle entanglement by three orders of magnitude (see also Results) (*22*).

**Fig. 1.**
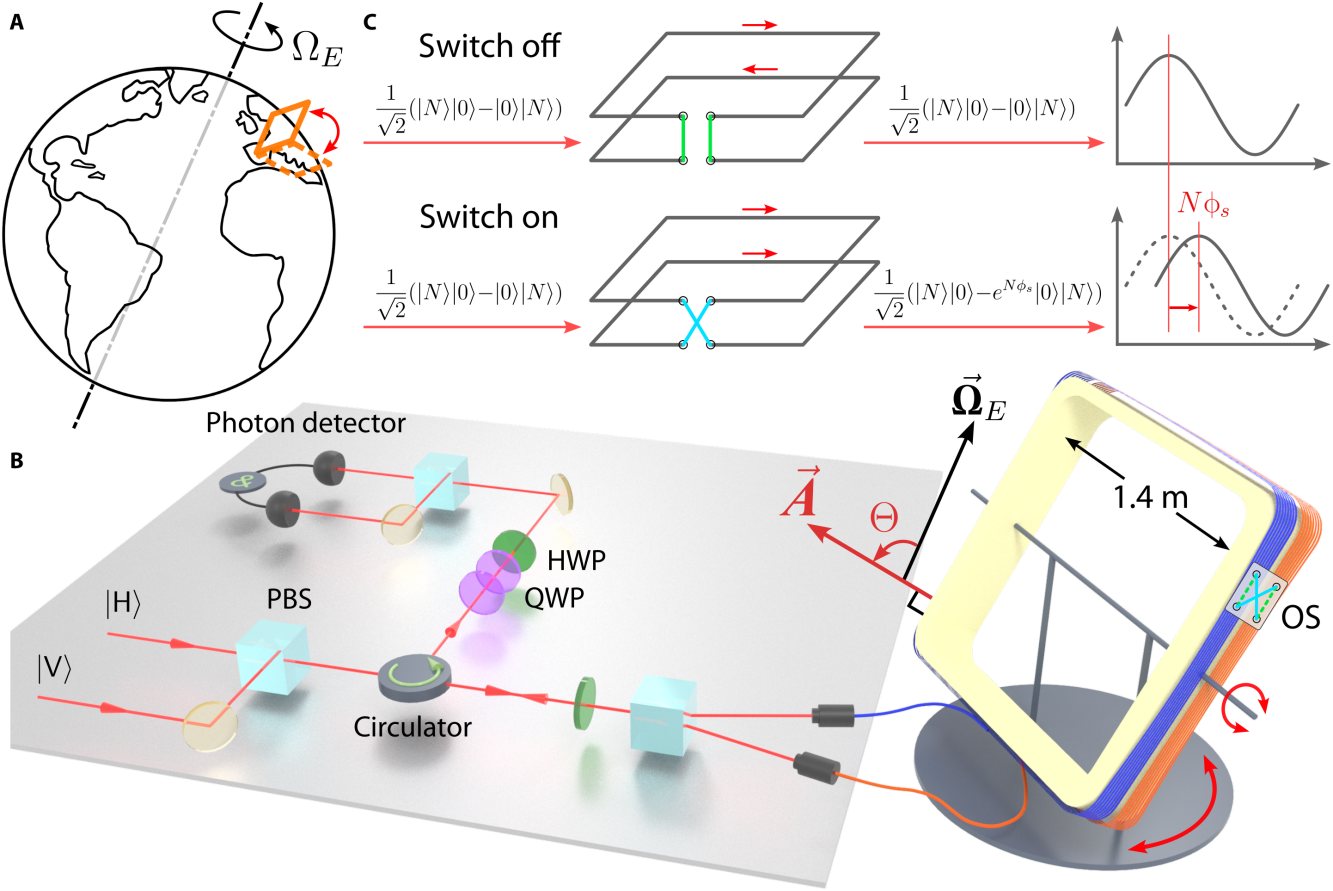
Earth’s rotation measured using entangled photons. (**A**) A rotatable 715-m^2^ Sagnac fiber interferometer is built in a laboratory located in Vienna, Austria. (**B**) Simplified schematic of the experimental setup. Orthogonally polarized photon pairs are converted to path-entangled N00N states in the Sagnac interferometer via a half-wave plate (HWP) followed by a polarizing beam splitter (PBS). The frame angle Θ is defined as the angle between Earth’s angular velocity vector ${\vec{\Omega }}_{E}$ and the fiber loop area vector $\vec{A}$ . The signal is extracted by observing the phase shift of quantum interference fringes induced by Earth’s rotation, using a set of quarter-wave plates (QWP) and a HWP, in combination with single-photon coincidence counting (&). (**C**) An optical switch (OS) is used to toggle Earth’s rotation signal on and off independent of the frame angle Θ. This is achieved by controlling the propagation direction (clockwise or counterclockwise) of photons in one half of the fiber spool.

We chose the detection of Earth’s rotation as a benchmark for our large-scale fiber interferometer, as its minute rate, fixed direction, and the absence of ways to manipulate its behavior make it particularly challenging to observe. On the other hand, the ubiquitous presence of acoustic- and seismic vibrations and thermal fluctuations transduce directly into phase noise in optical fiber (*37*) and drive the motion of the large apparatus. To solve these problems, we build our rotatable fiber interferometer with an optical switch to turn Earth’s rotation signal on and off, allowing us to fully characterize the angle-dependent Sagnac phase (Fig. 1).

According to the Sagnac effect (*38*, *39*), the flying times of photons traveling in opposite directions around a rotating encircled path are different, inducing a measurable phase difference

$$\phi_{s}=\frac{8\pi \Omega_{E}A\text{cosΘ}}{\lambda c}$$ (1)

Here, Ω*_E_* is the rotation angular frequency of Earth. *A* is the interferometer’s effective area of 715 m^2^ (for the calibration of the apparatus, see details in Materials and Methods). Θ is the angle between the area vector of the fiber loop and the angular velocity vector of Earth, and λ is the photon wavelength of 1546 nm.

In a Sagnac interferometer with a rotationally induced phase shift ϕ*_s_*, a two-mode coherent state ∣α⟩*_a_*∣α⟩*_b_* evolves to ∣α⟩*_a_*∣*e*^*i*ϕ*_s_*^α⟩*_b_*, where *a* and *b* are the two propagation directions in the interferometer and α is the complex amplitude of the state. After interfering on the beam splitter, the normalized intensity in the output arm α of the interferometer, previously used as the input, is *P_a_* = [1 + cos (ϕ*_s_*)]/2. We contrast this with multiphoton interference, which occurs when we inject the entangled state $\left({|N\rangle }_{a}{|0\rangle }_{b}+{|0\rangle }_{a}{|N\rangle }_{b}\right)/\sqrt{2}$ into the interferometer, where the *N* photons are in a superposition of being in either of the two modes. After propagating through the interferometer, the state evolves to $\left({|N\rangle }_{a}{|0\rangle }_{b}+e^{\text{iN}\phi_{s}}{|0\rangle }_{a}{|N\rangle }_{b}\right)/\sqrt{2}$ . At the interferometer output, the probability of finding exactly *N* photons in the output arm oscillates at *N* times the frequency: *P*_*N*,*a*_ ∝ [1 + cos (*N*ϕ*_s_*)] (*34*). For a single-photon input state, corresponding to *N* = 1, the detection probability coincides with the normalized intensity of coherent light. However, for *N* ≥ 2, an enhancement of the phase shift by a factor of *N* is observed.

## RESULTS

### Experimental implementation

The two-photon path-entangled state is realized by exploiting the polarization correlation of photon pairs emitted by a type-II spontaneous parametric down-conversion (SPDC) source (*40*). The photon pairs, centered at 1546 nm, are created in the product state ∣1⟩*_H_*∣1⟩*_V_*, where *H* and *V* denote horizontal and vertical polarization, respectively. A half-wave plate (HWP) oriented at 22.5° (with respect to the horizontal axis) transforms this product state into the polarization-entangled two-photon N00N state $\left({|2\rangle }_{H}{|0\rangle }_{V}-{|0\rangle }_{H}{|2\rangle }_{V}\right)/\sqrt{2}$ , where the cross terms cancel out because of the indistinguishability of the photons. Subsequently, this state is converted into a path-entangled state at a polarizing beam splitter (PBS), which separates the *H* and *V* photons into clockwise and counterclockwise propagating modes. After passing through the 2-km fiber loop, the clockwise-traveling photons pick up a Sagnac phase shift ϕ*_s_* induced by Earth’s rotation relative to the counterclockwise-traveling ones. The same PBS then converts the state back into the polarization-entangled state

$$\frac{1}{\sqrt{2}}\left({|2\rangle }_{H}{|0\rangle }_{V}-e^{i2\phi_{s}}{|0\rangle }_{H}{|2\rangle }_{V}\right)$$ (2)

Interference takes place again at the 22.5° HWP, leading to the output state

$$\frac{1}{\sqrt{2}}\text{sin}\phi_{s}\left({|2\rangle }_{H}{|0\rangle }_{V}+{|0\rangle }_{H}{|2\rangle }_{V}\right)-i\text{cos}\phi_{s}{|1\rangle }_{H}{|1\rangle }_{V}$$

A set of wave plates is used to control the detection probabilities by introducing a bias phase ϕ_0_. This artificially adds a relative phase between the *H* and *V* polarization components, allowing us to scan the full interference fringe and also project the measurements onto any polarization basis, turning the state in Eq. 2 into

$$\frac{1}{\sqrt{2}}\left[2_{H}0_{V}-e^{i\left(2\phi_{0}+2\phi_{s}\right)}0_{H}2_{V}\right]$$ (3)

To perform a projective measurement onto the ∣1⟩*_H_*∣1⟩*_V_* component of the state, we analyze the twofold coincidence probability *P_HV_* by collecting photons in both output ports of the PBS before the detectors

$$P_{\mathrm{HV}}=\frac{1}{2}\left[1+\text{cos}\left(2\phi_{0}+2\phi_{s}\right)\right]$$ (4)

This gives an enhancement factor of two in the observed Sagnac phase, as well as in the bias phase.

The central component of the Sagnac interferometer consists of 2-km fibers wound around a 1.4-m square aluminum frame (yellow; Fig. 1B). Because the detectable Sagnac phase shift induced by Earth’s rotation depends on the direction of the area vector $\vec{A}$ , the frame is designed to be rotatable in both pitch and yaw dimensions. This allows for a series of measurements to be taken at different values of Θ.

To more distinctly manifest the rotation signal, an optical switch is incorporated to toggle the effective area of the interferometer. The optical fiber is divided into two equal 1-km fiber segments (orange and blue), which are connected by the four-port optical switch. As shown in Fig. 1C, flipping the optical switch reverses the direction of light propagation in one of the fiber loops. When the optical switch is in the “OFF” state, the Sagnac phase shift is canceled out because of the opposite directions of light propagation in the two fiber segments, resulting in two area vectors with opposite signs and a zero effective area. By comparing the measurements in the optical switch “ON” and “OFF” states, it can be confirmed that the observed phase shifts are exclusively caused by Earth’s rotation.

**Fig. 2.**
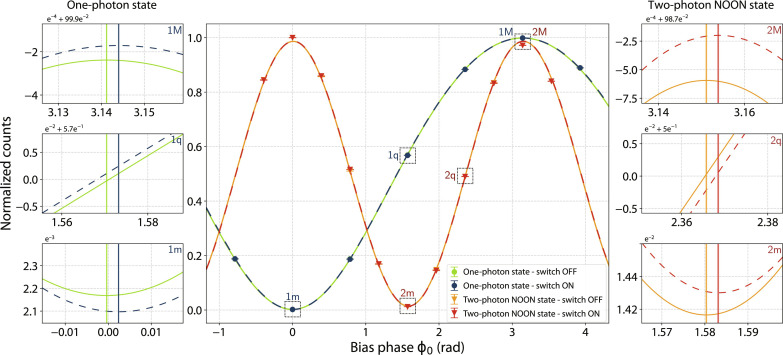
Quantum interference measurement revealing the Sagnac phase shift induced by Earth’s rotation. (**Middle**) Normalized quantum interference fringes of single-photon and two-photon entangled state measurements. The red and orange (blue and green) marks show the normalized two-photon (one-photon) coincidence counts with the Earth rotation signal switched on and off, respectively. The corresponding curves are least-squares fits to the data using a model of the experiment (see the Supplementary Materials). The doubled fringe frequency of the two-photon curves reveals the super-resolution due to quantum entanglement. (**Left** and **right**) Sagnac phase shifts induced by Earth’s rotation at Θ = 2.5°, zooming in around ϕ = π, π/2, and 0 for single-photon measurement (left) and around ϕ = π, 3π/4, and π/2 for two-photon measurement (right). The widths of the vertical lines indicate the size of uncertainties due to uncorrelated photon counting noise. Because the same phase bias ϕ_0_ has been applied to both one-photon and two-photon measurements, the doubled Sagnac phase shift does not manifest in the plots. 1, one-photon state; 2, two-photon N00N state; M, maximum; m, minimum; q, quadrature.

**Fig. 3.**
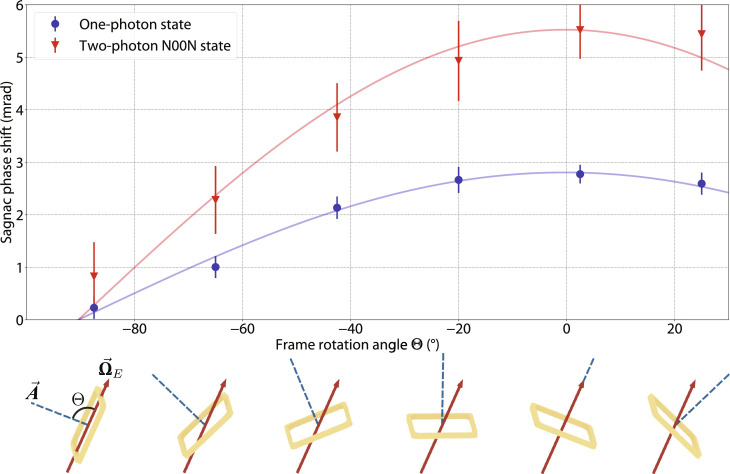
Sagnac phase shifts induced by Earth’s rotation measured at six interferometer frame angles. Θ’s range from −67.5° to +25°, evenly spaced by 22.5°. (**Top**) Each data point is obtained with the same measurement sequence and extraction method as Fig. 2. At each angle Θ, the Sagnac phase shift measured using two-photon entangled N00N states (red triangle marks) is found to be doubled compared with single-photon states (blue circle marks). The blue and red curves are the least-squares fits to Eq. 1 of the one-photon and two-photon N00N state measurements, respectively. (**Bottom**) Representation of different angles between the area vector of the interferometer (blue dashed line) and Earth’s rotation angular velocity vector (red arrow). The Sagnac phase shift induced by Earth’s rotation can be increased and decreased as the frame rotation angle is varied.

From Eq. 1, the Sagnac phase is maximized when the interferometer is oriented in a way that Earth’s rotation vector perpendicularly intersects the plane of the interferometer area. This orientation is determined from a calibration procedure with classical light in the interferometer (see details in Materials and Methods). Figure 2 shows the data for the Sagnac phase shifts induced by Earth’s rotation at Θ = 2.5°. The data points are acquired for one- and two-photon N00N states propagating through the interferometer. For the two-photon entangled states, 11 different data points were taken while continuously switching between the two operating modes: with and without Earth’s rotation signal (switch on and off, respectively). When alternating operation between the two modes at a frequency of 0.1 Hz, Earth’s rotation signal is resolved by comparing the interference fringes of the two modes. To further confirm that the phase shift is solely due to Earth’s rotation, additional data are acquired at various frame angles, thereby enabling curve fitting and precise phase difference extraction, as depicted in Fig. 3.

### Earth’s rotation-induced phase extraction

Figure 2 shows quantum interference fringes of the Sagnac interferometer at Θ = 2.5°. In the central figure, the red and orange marks represent normalized two-photon coincidence counts between the *H* and *V* photons measured with the optical switch on and off, respectively. These data were generated from 11 sets of 30-min contiguous integration periods. Each dataset was taken with a specific value of ϕ_0_, ranging from −π/8 to 2π + π/8 to cover a full interference fringe. The blue and green marks are heralded single-photon measurements, with 11 (7 shown) 15-min contiguous integration periods, ranging from −π/4 to 2π + π/4, serving as the reference measurement. The uncertainties for each data point are represented by ±1 SDs, which were calculated from Monte Carlo simulations using 10^5^ samples of Poisson-distributed photon coincidence counts (see details in Materials and Methods for a comprehensive error analysis).

For the two-photon measurements, the 11 data points obtained in each switch mode are fit to an interference fringe model. Earth’s rotation signal is extracted by calculating the phase shift between the two curves (red and orange). On the basis of Eq. 4, the data are fit with

$$N_{\text{switch off}}\left(\phi \right)=N_{0}\left[1+𝒱\cos\;(2\phi )\right]$$ (5)

$$N_{\text{switch on}}\left(\phi \right)=N_{0}\left[1+𝒱\cos\left(2\phi +\phi_{s}^{\left(2\right)}\right)\right]$$ (6)

where *N*_0_ is the amplitude of the photon interference, 𝒱 is the interference visibility, and $\phi_{s}^{\left(2\right)}$ is Earth’s rotation-induced phase shift to be measured. The extracted phase difference between two interference fringes is $\phi_{s}^{\left(2\right)}=5.5(5)\;\text{mrad}$ . A similar fitting and phase extraction procedure is used for single-photon reference measurements (blue and green), resulting in ϕ*_s_* = 2.8(2) mrad. In the two-photon measurement, the phase shift is enhanced by a factor of two because of the presence of two-photon path entanglement.

Sagnac phase shift measurements at five additional frame angles Θ are presented in Fig. 3. This plot explicitly shows two things: First, the Sagnac phase shift induced by Earth’s rotation is proportional to cos(Θ) as expected from Eq. 1. Second, the two-photon measurements consistently exhibit a doubled phase compared to the single-photon measurements for all the different frame angles. For each value of Θ, the Sagnac phase shift is extracted by comparing the interference fringes with the optical switch on and off, following a procedure identical to that used for Θ = 2.5°. The data for the five additional angles were acquired with a shorter integration time compared to Fig. 2, resulting in correspondingly larger statistical uncertainties. The red and blue curves are the least-squares fits using Eq. 1. From these fits, the maximum Sagnac phase shift induced by Earth’s rotation in the two-photon N00N state is 5.5(4) mrad, which corresponds to an Earth’s rotation rate of Ω*_E_* = 7.1(5) × 10^−5^ rad s^−1^, compared with 2.8(1) mrad or Ω*_E_* = 7.2(3) × 10^−5^ rad s^−1^ in the one-photon measurement. Both agree with the internationally accepted value 7.3 × 10^−5^ rad s^−1^ (*41*). The experimentally determined enhancement factor due to two-photon quantum entanglement is 1.96(15).

**Fig. 4.**
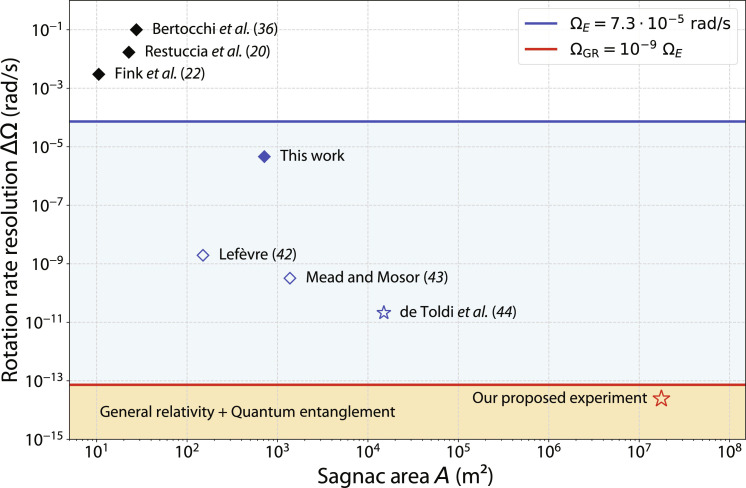
Rotation rate resolutions and enclosed areas of existing and predicted quantum optical Sagnac interferometers. The plot is divided into three sensitivity regimes: sensitivity below Earth rotation Ω*_E_* (white zone), sensitivity above Ω*_E_* but below general relativistic effects Ω_GR_ (blue zone), and sensitivity above Ω_GR_ (orange zone). Diamond markers represent existing interferometric platforms, while star markers are proposed platforms but yet to be realized. Solid markers represent performed experiments with quantum states of light, while empty markers represent experiments yet to be performed. Bertocchi *et al.* (*36*): *L_f_* = 550 m and *P* = 0.63 m; Restuccia *et al.* (*20*): *L_f_* = 100 m and *P* = 2.85 m; Fink *et al.* (*22*): *L_f_* = 270.5 m and *P* = 0.49 m; this work: *L_f_* = 2 km and *P* = 5.6 m; Lefèvre (*42*): *L_f_* = 3 km and *P* = 0.63 m; Mead and Mosor (*43*): *L_f_* = 8 km and *P* = 2.15 m; de Toldi *et al.* (*44*): *L_f_* = 15 km and *P* = 12.57 m; our proposed experiment: *L_f_* = 47.5 km and *P* = 6 km, where *L_f_* is the fiber length and *P* is the perimeter. The photon pair generation rate is 1 GHz for the (*42*, *43*) and 10 GHz for the (*44*) and our proposed experiment, with integration times on the order of a month (for more details see the Supplementary Materials).

The achieved phase resolution in our experiment is primarily hindered by scale factor instability, with the most detrimental contributions coming from mechanical vibrations of the frame due to its extensive surface area, thermal fluctuations, and acoustic noise. Scaling to larger interferometric areas will be possible by incorporating design lessons from cutting-edge fiber-optic gyroscopes (FOGs). FOGs using classical light have reached phase resolutions of less than a nanoradian, translating to rotation rates below 0.1 nrad s^−1^, with a stable signal over more than a month (*42*, *43*). Combining proposals for next-generation FOGs, such as the Giant-FOG with an area of 15,000 m^2^ (*44*), with state-of-the-art single-photon sources (*45*), we anticipate that a phase resolution of about 20 prad s^−1^ could be reached with quantum states of light, which is within two orders of magnitude of the general-relativistic rotation rate correction term Ω_GR_ = 10^−9^ Ω*_E_* because of the frame-dragging and the geodetic effect (see Fig. 4) (*46*, *47*).

## DISCUSSION

We have demonstrated the largest and most precise quantum-optical Sagnac interferometer to date, exhibiting sufficient sensitivity to measure Earth’s rotation rate. Our work advances the state of the art of entanglement-based rotation sensors by three orders of magnitude and introduces a signal-switching technique that can be used to modulate the effective area of the interferometer. This enables a self-referenced measurement of the fixed-rate rotation signal, without the need for building an additional small-area interferometer (*48*), thereby going beyond previous work focusing on sensing an induced motion relative to the surrounding laboratory.

When comparing the use of two-photon entangled states to single-photon states, we observe an improvement by a factor of two in the measured phase value due to super-resolution. Our approach using a polarization encoding is readily scalable to N00N states with higher photon numbers (*34*), with the main limitations being the large amount of transmission loss of the experimental setup and the generation of the multiphoton states.

Our methods pave the way for other technically challenging proposals, such as dynamically generating entanglement from the underlying space-time (*48*), directly probing gravitationally induced phase shifts (*49*, *50*), rotational and gravitational decoherence in the quantum interference of photons (*51*), testing fundamental symmetries in quantum field theory (*52*), investigating local Lorentz invariance violation (*53*), detecting exotic low-mass fields from high-energy astrophysical events (*54*), and dark matter searches such as axion-photon coupling (*52*). It is predicted that two photons can transition to axions in the presence of an external magnetic field. In an optical Sagnac interferometer, where two orthogonal polarizations counterpropagate, the component parallel to the magnetic field would then be retarded with respect to the other, leading to a nonreciprocal observable phase shift or the loss of two photons. Our interferometer constitutes an excellent testbed for this phenomenon, allowing investigations both with classical light and entangled photons.

In conclusion, the successful observation of Earth’s rotation using entangled states of light, a century after the first local observation of Earth’s rotation-induced fringe shift with a Sagnac interferometer (*2*), constitutes a milestone toward the goal of probing the interface between quantum mechanics and general relativity. The zero-area switching technique that we have introduced, which allows the rotation signal to be referenced to an effectively nonrotating frame, is a key technical advancement over previous works (*20*). This is manifested in the greatly improved sensitivity over previous entanglement-based sensors, which, in turn, shows the promise of our approach for measuring general-relativistic non-inertial effects on quantum states.

## MATERIALS AND METHODS

### Characteristics of the experimental setup

A detailed experimental setup is provided in fig. S1. A periodically poled KTiOPO_4_ crystal produces orthogonally polarized photon pairs centered at 1545.76 nm in a type-II SPDC process. The crystal is pumped by continuous wave (CW) Ti:Sapphire laser (Coherent Mira HP) emitting at 772.88 nm (*40*). The photon source pump power is set to 145 mW, leading to a detected photon pair coincidence rate of approximately 400 kHz. The photons in each generated pair are combined on a PBS and are overlapped temporally using a delay line in one of the input ports of the PBS. The total loss of the entire experimental setup is 90% (10 dB). The Sagnac loop introduces 5 dB of losses, out of which 1 dB is the optical switch insertion loss, 1 dB from the 2-km polarization-maintaining (PM) fiber (≈0.5 dB/km), and 3 dB from fiber connections, while the input and output of the optical setup contribute the remaining 5 dB. In detection, photons from the output paths are coupled into single-mode fibers connected to superconducting nanowire single-photon detectors, housed in a 1 K cryostat, with a detection efficiency of roughly 95% and a dark-count rate around 300 Hz. Amplified detection signals are counted using a time tagging module with a timing resolution of 156.25 ps, and two-photon coincidence events are extracted using a coincidence window of 3.75 ns. In the two-photon N00N state measurements, when both photons are propagating through the interferometer, the detected photon pair rate is around 4 kHz, consistent with the expected exponential fragility to losses of a two-photon N00N state $1-\eta_{i}^{2}\approx 99\%$ , where η_i_ = 0.1 is the total transmission efficiency of the interferometer. In the one-photon measurements, one photon of the pair is used as a trigger while the other propagates through the interferometer. The total heralded single-photon rate in the two detection ports is around 20 kHz, which is compatible with the overall losses 1 − η_t_η_i_ ≈ 95%, where η_t_ = 0.5 is the transmission coefficient of the trigger photon fiber path.

### Interferometer calibration

**Fig. 5.**
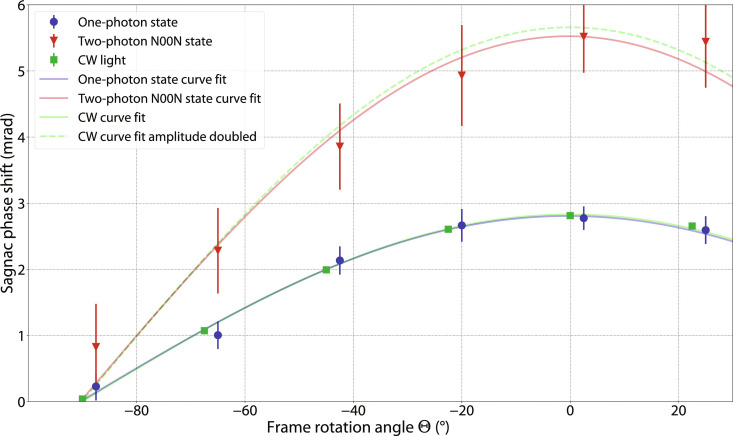
Comparison of photon measurements and CW measurements as calibration. Data of one-photon state (blue) and two-photon N00N state data (red) are inherited from Fig. 3. Green square marks are obtained using a polarimeter with CW light at six different Θ’s ranging from −90° to +22.5°, evenly spaced by 22.5°. The green solid curve is the least-squares fit of CW measurements with fitting function as ϕ*_E_*(Θ) = *S*Ω*_E_* cos (Θ + Θ_0_). This measurement allows us to find the frame angle that maximizes the Sagnac phase (Θ = 0°). The green dashed curve is plotted as 2*S*Ω*_E_* cos (Θ + Θ_0_) to compare with the two-photon N00N state measurements.

In the laboratory, the axis normal to the fiber spool plane when vertically oriented with respect to the horizon is pointed north. The rotational degree of freedom of the fiber loop frame introduces the opportunity for experimental calibration of the interferometer by estimating its scale factor S, while assuming Earth’s rotation rate (Ω*_E_*) as a known quantity, with ϕ*_s_* = *S*Ω*_E_*. A set of phase measurements are performed with a CW light source at telecom wavelength at six different angular positions Θ*^k^* of the fiber loop frame spaced by 22.5° (see Fig. 5), allowing us to find the frame angle that maximizes the Sagnac phase (Θ = 0°). H-polarized light is injected into the interferometer, which is converted into diagonal polarization by a HWP before entering the Sagnac interferometer. Because of Earth’s rotation, the *H* and *V* components acquire a relative Sagnac phase ϕ*_s_*, which is encoded in the polarization state ellipticity angle χ (*55*), such that ϕ*_S_* = 2χ. A compact free-space polarimeter is used to fully characterize the polarization state after the wave plates, which compensate first for the polarization rotation in the output fiber circulator path (see fig. S2). As in the measurements with quantum light, the optical switch is driven by a 0.1-Hz square wave. The recorded time trace of χ is partitioned into two sets by demodulating it using the driving signal. For each frame angle Θ*^k^*, the differential average between the two traces $\delta {\bar{\chi }}^{k}={\bar{\chi }}_{\text{on}}^{k}-{\bar{\chi }}_{\text{off}}^{k}$ is used to calculate the Sagnac phase $\phi_{S}^{k}$ and its associated uncertainty σ*^k^*. As part of a Monte Carlo simulation resampling the phase values $\phi_{S}^{k}$ using their uncertainties, the data are fit to the model function ϕ*_E_*(Θ) = *S*Ω*_E_* cos (Θ + Θ_0_), where Ω*_E_* = 7.29 × 10^−5^ rad s^−1^ is the known value of Earth’s rotation rate and *S* and Θ_0_ are free parameters. The Monte Carlo simulation estimating these parameters additionally samples the frame angles Θ*^k^* from uniform probability distributions [Θ*^k^* − 1, Θ*^k^* + 1]. The extracted fit parameters are the scale factor *S* = 38.8(1) and the angular offset Θ_0_ = 0.03(33)°. The CW measurements are compared with the photon measurements in Fig. 3.

### Noise mitigation

The interferometer frame is fixed on an air-floated optical table to dampen the transduction of ambient seismic vibrations into the frame. The fiber spools are covered with layers of insulation material (Thinsulate) to mitigate temperature- and air current-induced spatial gradients and time-varying fluctuations of the fiber length and refractive index. This passive isolation increases the scale factor stability in time by stabilizing the enclosed interferometric area.

More crucially, the optical switching method is also a fundamental and powerful tool in our experimental implementation. By zeroing the interferometer’s effective area, we are able to obtain a reference measurement, allowing for the distinction and elimination of spurious signals arising from various technical and background noise sources, which include laser intensity fluctuations, imperfections in the input photon state, nonideal polarization rotations during light propagation out of the fiber loop, and variations in mechanical stresses applied to the frame structure across its angular orientations. Furthermore, the modulation of the signal at a specific frequency helps mitigate slow frequency drifts in the measured phase via postprocessing, thereby increasing its long-term stability over acquisition times spanning hours.

### Phase estimation and uncertainty analysis

The phase shifts and associated uncertainties presented in Fig. 3 are estimated using a Monte Carlo simulation accounting for photon counting noise and uncertainties in the phase offset. In each round of the simulation, the photon counts are sampled using a Poisson distribution with mean and SD of $N_{k}^{s}$ and $\sqrt{N_{k}^{s}}$ , respectively, where $N_{k}^{s}$ is the number of recorded photon counts for the offset phase $\phi_{0}^{k}$ and switch state *s* ∈ {on, off}. In addition, phase-offset noise, correlated between the on and off states, is sampled from a distribution derived from the waveplate motor repeatability and added to the offsets $\phi_{0}^{k}$ . For each sampled dataset, a least-squares fit is performed, using the amplitude *N*_0_, fringe visibility 𝒱, and phase shift ϕ as free parameters. Last, the values and uncertainties of these parameters are estimated using the mean and SD, respectively, taken over 10^5^ repetitions of the simulation.

## Supplementary Materials

### This PDF file includes:

Supplementary Text Figs. S1 and S2 Tables S1 to S3 References
