# Supplementary material for: Experimental observation of Earth’s rotation with quantum entanglement
Source: Sci Adv. 2024 Jun 14;10(24):eado0215. doi: 10.1126/sciadv.ado0215 (PMC11177943; doi:10.1126/sciadv.ado0215)
Supplement: Supplementary file 1 — Supplementary Text Figs. S1 and S2 Tables S1 to S3 References [file sciadv.ado0215_sm.pdf]

Science Advances
AAAS

Supplementary Materials for

Experimental observation of Earth's rotation with quantum entanglement

Raffaele Silvestri *et al.*

Corresponding author: Haocun Yu, haocun.yu@univie.ac.at; Philip Walther, philip.walther@univie.ac.at

Sci. Adv. **10**, eado0215 (2024)
DOI: 10.1126/sciadv.ado0215

This PDF file includes:

Supplementary Text
Figs. S1 and S2
Tables S1 to S3
References

Interferometer with classical light:

Continuous-wave (CW) light source: The CW source employed is a broadband NIR-wavelength Superluminescent Diode (SLD). In front, a hard-coated bandpass filter with 12 nm full-width at half-maximum (FWHM) centered at 1545.5 nm is placed.

Interferometer operation: The light enters horizontally polarized (H), is coupled into a single-mode (SM) fiber-optic circulator, and turns diagonally polarized (+) after a half-wave plate (HWP) oriented at 22.5° with respect to H. A Wollaston prism (WP) serves as a high-extinction ratio polarizing beam-splitter (PBS), which spatially separates the light into its horizontal and vertical (V) components. The output beams of the WP are coupled into polarization-maintaining (PM) fiber, aligned so that both polarization components propagate in the slow axis of the fiber. This ensures that the birefringence of the fiber does not contribute to the phase noise. A four-port PM optical micro-electro-mechanical systems (MEMS) switch connects the two equal fiber spools of 1 km length. When this switch is in the state "ON", the H and V components pick a relative non-reciprocal Sagnac phase shift ϕ_S. After light recombination at the PBS, the polarization ellipse major axis turns to anti-diagonal (azimuth angle $\psi = -45°$), while being slightly elliptical (ellipticity angle $\chi = \phi_S/2$). When passing the same HWP again, the polarization ellipse rotates by 45° with its major axis back to horizontal while still maintaining its ellipticity ($\psi = 0, \chi = \phi_S/2$), then undergoes the fiber circulator and is coupled back to free space. It follows a set of 3 waveplates, respectively two quarter-wave plates (QWP) and one HWP, with a second WP as detection PBS (see Fig. S1) *(56, 57)*.

Waveplate operation: A set of two quarter-wave and one half-wave plate can be used to implement any polarization transformation *(58)*. Thus, such a set of waveplates is able to perform several tasks at the same time.
1) Polarization state tomography: They can select any polarization basis for the measurement, namely rectilinear $\{H, V\}$, diagonal $\{+, -\}$ ($\{D, A\}$) and circular $\{RL\}$, with their corresponding unitary operators to set the measurement basis being $\{I, \hat{\sigma}_2, \hat{\sigma}_3\}$.
2) Polarization compensation of the circulator fiber: Since the SM fiber of the circulator introduces random birefringence, the polarization-state rotation at the output needs to be compensated for. This can be achieved by preparing the state before the fiber in two different known polarization states, for instance with two separate H and + polarizers. If full polarization tomography on the two output states $|P_H\rangle = U_f |H\rangle$, $|P_+\rangle = U_f |+\rangle$ is performed, the fiber unitary U_f can be fully reconstructed. By configuring the waveplates to implement the inverse matrix U_f^{-1} one reduces the overall action of the circulator on the polarization state to an identity transformation.
3) Selecting the working point: To set the working point of the interferometer it is sufficient to apply a unitary $U(\phi) = e^{-i\phi\hat{n}_2\cdot\vec{\sigma}/2}$ that rotates the compensated polarization state along the diagonal axis $\hat{n}_2$ by an angle ϕ, which corresponds to the bias phase. The rotation operator can

then simply be written as a phase shift matrix

$$U(\phi) = \begin{pmatrix} 1 & 0 \\ 0 & e^{i\phi} \end{pmatrix} \tag{S1}$$

expressed in the diagonal basis

$$U_{\text{bias}}(\phi) = U_{\text{HWP}}(-22.5°)U(\phi)U_{\text{HWP}}(-22.5°), \tag{S2}$$

where $U_{\text{HWP}}(-22.5°)$ is the unitary of an HWP oriented at -22.5°. The operation physically corresponds to rotating the compensated H polarization to A, applying a relative phase between the H and V components, and rotating back.
In the end, the waveplates are able to implement an overall unitary $U = U_{\text{proj}}U_{\text{bias}}U_f^{-1}$, with $U_{\text{proj}} \in \{I, \hat{\sigma}_2, \hat{\sigma}_3\}$, by setting a triplet of angles $(\theta_1, \theta_2, \theta_3)$ such that

$$U = U_{\text{HWP}}(\theta_3)U_{\text{QWP}}(\theta_2)U_{\text{QWP}}(\theta_1). \tag{S3}$$

Phase extraction: To measure the Sagnac phase encoded in the polarization state after the waveplates, a compact free-space polarimeter with a sampling rate of 20 Hz is employed. The phase is estimated as $\phi_S \approx 2\sqrt{\overline{\delta\chi}^2 + \overline{\delta\psi}^2}$, for small differential mean ellipticity $\overline{\delta\chi}$ and azimuth $\overline{\delta\psi}$ angles, where δ denotes the difference across the two switch states. Ideally, the Sagnac phase should only be encoded in the ellipticity angle. However, due to imperfect polarization rotation from the waveplates, the measurement bases will change, and a small part of the phase signal couples into the azimuth. The employed expression represents then a good approximation of the effective Sagnac phase value for small angular shifts.

Optical switch operation: A square-wave modulation at 0.1 Hz with a 50 % duty cycle was employed for all the measurements (classical and quantum). Even though in principle our four-port PM fiber optical MEMS switch could operate at a modulation signal frequency of almost 1 kHz, we observed a noisy transition region of 20 ms duration centered at each rising/falling edges of the square signal. For this reason, in post-processing, photon counts within a 10 ms time window from to the edges are discarded to make sure none of the time tags fall in a noisy region, while for the CW measurement at least the two data samples around the transitions are removed (50 ms time window) due to the limited sampling rate (20 Hz) of the polarimeter (see Fig. S2). This procedure effectively limits the switching frequency to less than 25 Hz. In the end, a modulation at 0.1 Hz was selected since it showed the best stability over time. The optical switch also introduces a state-dependent power loss of 10 % when is turned off, so that the transmission in the “OFF” state is 0.9 of the “ON” state.

Interferometer with quantum light:

Single-photon state measurement: A HWP oriented at 45° is placed into the V photon input path, the photon is thus transmitted by the PBS and directly coupled into a single-mode fiber

connected to a detector channel, serving as a trigger, while the H photon is transmitted out of the other port and injected into the interferometer (see Fig. S1). Two-photon coincidence counts between the two output channels and the trigger are recorded, and for each phase bias the total number of events of each switch state is $N_H^{(\text{on/off})}$. The selected observable for the fringe scan points is the total normalized V counts

$$n_V = \frac{N_V}{N_H + N_V}, \tag{S4}$$

where

$$N_H = \frac{A_H}{2}[1 + \mathcal{V}_H \cos(\phi + \phi^{(1)})], \tag{S5}$$

$$N_V = \frac{A_V}{2}[1 - \mathcal{V}_V \cos(\phi - \phi^{(1)})] \tag{S6}$$

with the constraints $\mathcal{V}_H = \mathcal{V}_V = \mathcal{V}$ and $\phi_H^{(1)} = \phi_V^{(1)} = \phi^{(1)}$. The corresponding fitting function is

$$n_V(\phi) = a_V \frac{1 - \mathcal{V}\cos(\phi + \phi^{(1)})}{1 + \eta\mathcal{V}\cos(\phi + \phi^{(1)})}, \tag{S7}$$

where $a_V = A_V/(A_H + A_V)$ and $\eta = (A_H - A_V)/(A_H + A_V)$. The fringe visibility $\mathcal{V}$, phase offset $\phi^{(1)}$ as well as the amplitudes A_H and A_V are taken as free parameters of the fit.

N00N state measurement: By removing or rotating the HWP to 0°, and adjusting the relative temporal delay to make the H and V photons indistinguishable in all other degrees of freedom, a $|1_H\rangle|1_V\rangle$ Fock state is generated after the input PBS. The Hong-Ou-Mandel interference in polarization takes place at the 22.5° HWP, generating the two-photon N00N state. Coincidence photon counts between the two output channels are recorded, for each phase bias, and the total number of photons in the two switch states $N_{HV}^{(\text{on/off})}$ are counted. We select the latter quantity as observable for the fringe scan. The fitting function is

$$N_{HV}(\phi) = \frac{A_{HV}}{2}[1 + \mathcal{V}\cos(2\phi + \phi^{(2)})], \tag{S8}$$

with A_{HV}, $\mathcal{V}$, and $\phi^{(2)}$ as free parameters.

Sensitivity analysis of proposed experiments:

For the analysis of the proposed experiments with two-photon NOON states (empty markers), the Heisenberg phase precision scaling at the point of maximum sensitivity is assumed $\delta\phi = 1/2\sqrt{R_{out}T}$, where R_{out} is the detected photon pairs rate and T is the total integration time of

the measurement. The newly proposed switching technique is included and $\delta\phi_{on} = \delta\phi_{off} = \delta\phi$ is imposed with $T/2$ integration time for each state, giving

$$\delta\phi = \frac{1}{\sqrt{2R_{out}T}}. \tag{S9}$$

The optical losses during propagation in fiber are exponential in its length, thus the output rate is related to the input rate as $R_{out} = \eta R_{in}$, with transmission coefficient $\eta = 10^{-\alpha L_f/10}$, where α and L_f are the fiber attenuation coefficient and length, respectively. For a general NOON state the losses are exponential in the number of photons N such that $\eta \rightarrow \eta^N$, so the two-photon rate is

$$R_{out} = 10^{-2\frac{\alpha L_f}{10}} R_{in}. \tag{S10}$$

In the end, the phase precision is given by

$$\delta\phi = \frac{1}{\sqrt{2R_{in}T}} 10^{\frac{\alpha L_f}{10}}. \tag{S11}$$

The corresponding rotation rate precision is then given by the Sagnac phase formula at maximum signal $\delta\Omega = \delta\phi/S$, with the scale factor defined as $S = \frac{8\pi A}{\lambda c}$, where λ is the light wavelength and A is the effective area of the interferometer. $A = A(L_f)$ is also a function of the fiber length depending on the interferometer geometry. In particular, for the proposed giant square ring interferometer (GFRING) the effective area is $A = \frac{1}{n_t}(\frac{L_f}{4})^2$, where n_t are the number of fiber turns around the square frame. Given the expected dimensions of the ring it is assumed that its frame plane would be fixed parallel to the Earth surface at its location. The rotation rate resolution can then be written as

$$\delta\Omega = \sqrt{\frac{2}{R_{in}T}} \frac{\lambda c}{\pi \sin\theta_L} n_t \frac{10^{\frac{\alpha L_f}{10}}}{L_f^2}, \tag{S12}$$

where θ_L is the latitude at the location of the ring. The geometrical parameters of the GFRING are numerically extracted by requiring the signal-to-noise ratio (SNR) to be $\Omega_{GR}/\delta\Omega = 3$ and maximizing n_t to reduce the surface area of the ring as much as possible. The calculation assumes the latitude angle $\theta_L = 48.2°$ of Vienna, Austria. Finally, the optimal fiber length and number of turns result in $L_f = 47.5$ km and $n_t = 8$, corresponding to a square side of 1.5 km and a perimeter of 6 km. For each experiment an integration time of $T = 2$ months is considered (see Table S3).

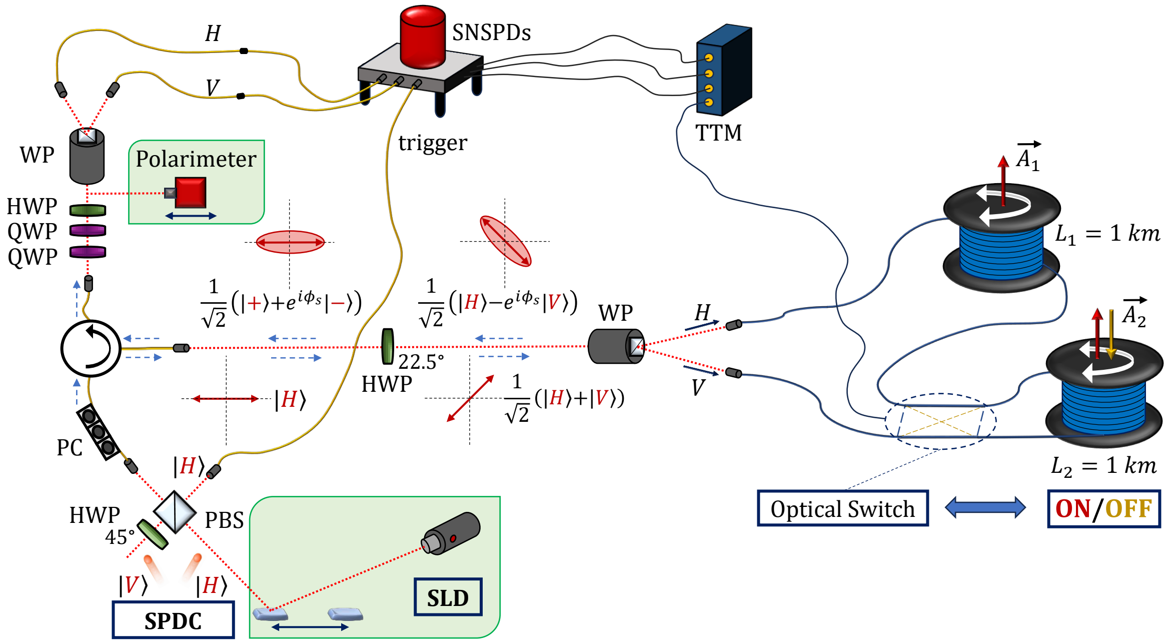

Fig. S1. Detailed experimental setup. Experimental scheme for the single-photon and CW measurements (green insets). In the one-photon measurement a HWP at 45° before the input PBS is used to send the trigger photon directly to the SNSPDs. For the CW measurement the SNSPDs are replaced with a polarimeter placed after the waveplates. The polarization states before/after the Sagnac are indicated with red arrows, and the Earth rotation phase manifests as ellipticity in the output polarization state. SPDC: spontaneous parametric down-conversion single photons source. SLD: superluminescent diode. PBS: polarizing beam splitter cube. PC: fiber polarization controller. WP: Wollaston prism. HWP: half-wave plate. QWP: quarter-wave plate. SNSPDs: superconducting nanowire single-photon detectors. TTM: time tagging module.

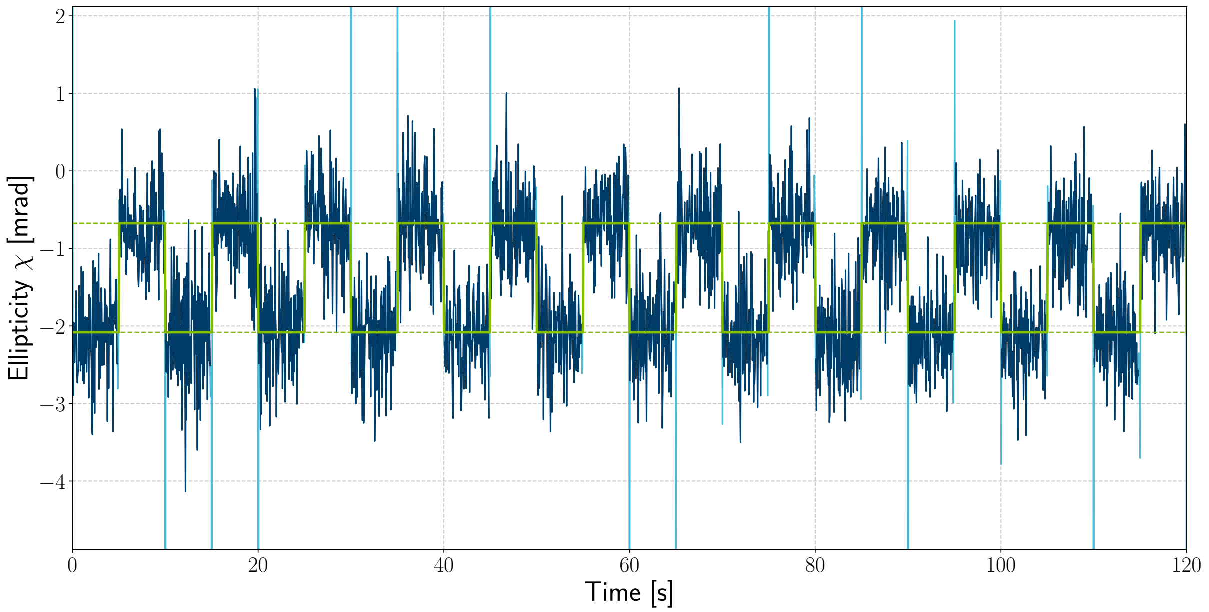

Fig. S2. Ellipticity angle time signal. The ellipticity angle measured with CW light by a polarimeter, with 20 Hz sampling rate, is plotted over time. It is shown how the square-wave voltage applied to the switch at 0.1 Hz modulates the measured signal (dark blue trace). The high/low levels correspond to the switch on/off states (green square wave). Data samples in the trace are cut around the transition points (light blue samples) and two separate on/off time traces are extracted. $\delta\overline{\chi}$ is calculated as the difference between the two respective averages $\overline{\chi}_{on} - \overline{\chi}_{off}$ (green dashed horizontal lines). The same analysis is performed on the azimuth time trace.

Tables

Θ	$\mathcal{V}_{\text{on}}$	$\mathcal{V}_{\text{off}}$	ϕ_{on} (mrad)	ϕ_{off} (mrad)	ϕ_{E} (mrad)
$-87.5°$	99.66(12) %	99.67(12) %	-1.36(2.45)	-1.13(2.45)	0.23(21)
$-65°$	99.68(12) %	99.69(12) %	-1.99(2.45)	-0.98(2.45)	1.00(21)
$-42.5°$	99.69(12) %	99.68(12) %	-1.86(2.45)	0.28(2.45)	2.14(21)
$-20°$	99.59(13) %	99.60(13) %	-7.80(2.45)	-5.13(2.45)	2.66(25)
$2.5°$	99.69(13) %	99.67(13) %	-2.43(2.43)	0.34(2.46)	2.77(18)
$25°$	99.66(12) %	99.66(12) %	-1.22(2.45)	1.37(2.45)	2.59(21)

Table S1. Single-photon state measurement extracted parameters. Interferometric visibility $\mathcal{V}_{\text{on/off}}$ and phase offset $\phi_{\text{on/off}}$ values extracted from the fit for each frame orientation angle Θ both in the switch on and off states, with the corresponding Earth rotation induced phases ϕ_{E} calculated as $\phi_{\text{off}} - \phi_{\text{on}}$.

Θ	$\mathcal{V}_{\text{on}}$	$\mathcal{V}_{\text{off}}$	ϕ_{on} (mrad)	ϕ_{off} (mrad)	ϕ_{E} (mrad)
$-87.5°$	96.81(45) %	96.79(45) %	-28.53(4.94)	-27.71(4.95)	0.82(65)
$-65°$	96.78(45) %	96.73(45) %	-21.76(4.93)	-19.49(4.94)	2.28(65)
$-42.5°$	96.72(45) %	96.56(45) %	-9.87(4.94)	-6.02(4.95)	3.86(65)
$-20°$	95.67(44) %	95.56(44) %	-5.00(4.95)	-0.07(4.96)	4.93(76)
$2.5°$	97.14(45) %	97.17(45) %	-24.60(4.92)	-19.09(4.92)	5.51(54)
$25°$	97.54(46) %	97.51(46) %	-10.53(4.94)	-5.09(4.95)	5.44(69)

Table S2. Two-photon NOON state measurement extracted parameters.

	L_f	P	A	S (s^{-1})	$\delta\phi_S$ (rad)	$\delta\Omega$ (rad s^{-1})
□ This work	2 km	5.55 m	715 m^2	38.8	$1.15 \cdot 10^{-4}$	$4.61 \cdot 10^{-6}$
○ Lefèvre et al. *(42)*	3 km	0.63 m	150 m^2	8.1	$1.34 \cdot 10^{-8}$	$1.95 \cdot 10^{-9}$
○ Mead et al. *(43)*	8 km	2.15 m	1372 m^2	74	$2.38 \cdot 10^{-8}$	$3.21 \cdot 10^{-10}$
○ de Toldi et al. *(44)*	15 km	12.57 m	15000 m^2	811	$1.69 \cdot 10^{-8}$	$2.11 \cdot 10^{-11}$
□ Our proposed experiment	47.5 km	6 km	17.6 km^2	951320	$2.31 \cdot 10^{-8}$	$2.43 \cdot 10^{-14}$

Table S3. Quantum optical Sagnac interferometers specifications and resolutions comparison. Square markers (□) represent square interferometer frames, while (○) are circular frames. The selected optical wavelength λ is 1550 nm for each proposed experiment. The FOGs platforms utilize PM fibers (α = 0.5 dB/km) in *(42, 43, 44)*, while we propose to use a standard single-mode fiber (α = 0.16 dB/km) for our future experiment. L_f is the fiber length, P is the frame perimeter, A is the effective interferometer area, S is the scale factor, while $\delta\phi_S$ and $\delta\Omega$ are the Sagnac phase and rotation resolutions.

REFERENCES AND NOTES

1. A. A. Michelson, E. W. Morley, On the relative motion of the Earth and the luminiferous ether. *Am. J. Sci.* **34**, 333–345 (1887).

2. A. A. Michelson, H. G. Gale, The effect of the Earth's rotation on the velocity of light. *ApJ* **61**, 137 (1925).

3. LIGO Scientific Collaboration and Virgo Collaboration, Observation of gravitational waves from a binary black hole merger. *Phys. Rev. Lett.* **116**, 061102 (2016).

4. J. E. Zimmerman, J. E. Mercereau, Compton wavelength of superconducting electrons. *Phys. Rev. Lett.* **14**, 887–888 (1965).

5. F. Hasselbach, M. Nicklaus, Sagnac experiment with electrons: Observation of the rotational phase shift of electron waves in vacuum. *Phys. Rev. A* **48**, 143–151 (1993).

6. S. A. Werner, J. L. Staudenmann, R. Colella, Effect of Earth's rotation on the quantum mechanical phase of the neutron. *Phys. Rev. Lett.* **42**, 1103–1106 (1979).

7. F. Riehle, T. Kisters, A. Witte, J. Helmcke, C. J. Bordé, Optical Ramsey spectroscopy in a rotating frame: Sagnac effect in a matter-wave interferometer. *Phys. Rev. Lett.* **67**, 177–180 (1991).

8. A. Lenef, T. D. Hammond, E. T. Smith, M. S. Chapman, R. A. Rubenstein, D. E. Pritchard, Rotation sensing with an atom interferometer. *Phys. Rev. Lett.* **78**, 760–763 (1997).

9. T. L. Gustavson, A. Landragin, M. A. Kasevich, Rotation sensing with a dual atom-interferometer Sagnac gyroscope. *Class. Quantum Grav.* **17**, 2385–2398 (2000).

10. J. K. Stockton, K. Takase, M. A. Kasevich, Absolute geodetic rotation measurement using atom interferometry. *Phys. Rev. Lett.* **107**, 133001 (2011).

11. R. Gautier, M. Guessoum, L. A. Sidorenkov, Q. Bouton, A. Landragin, R. Geiger, Accurate measurement of the Sagnac effect for matter waves. *Sci. Adv.* **8**, eabn8009 (2022).

12. K. Schwab, N. Bruckner, R. E. Packard, Detection of the Earth's rotation using superfluid phase coherence. *Nature* **386**, 585–587 (1997).

13. R. Simmonds, A. Marchenkov, E. Hoskinson, J. Davis, R. Packard, Quantum interference of superfluid 3He. *Nature* **412**, 55–58 (2001).

14. S. Gupta, K. W. Murch, K. L. Moore, T. P. Purdy, D. M. Stamper-Kurn, Bose-einstein condensation in a circular waveguide. *Phys. Rev. Lett.* **95**, 143201 (2005).

15. S. Levy, E. Lahoud, I. Shomroni, J. Steinhauer, The a.c. and d.c. Josephson effects in a Bose-Einstein condensate. *Nature* **449**, 579–583 (2007).

16. G. E. Marti, R. Olf, D. M. Stamper-Kurn, Collective excitation interferometry with a toroidal Bose-Einstein condensate. *Phys. Rev. A* **91**, 013602 (2015).

17. H. Lee, P. Kok, J. P. Dowling, A quantum rosetta stone for interferometry. *J. Mod. Opt.* **49**, 2325–2338 (2002).

18. V. Giovannetti, S. Lloyd, L. Maccone, Quantum metrology. *Phys. Rev. Lett.* **96**, 010401 (2006).

19. M. Jaffe, P. Haslinger, V. Xu, P. Hamilton, A. Upadhye, B. Elder, J. Khoury, H. Müller, Testing sub-gravitational forces on atoms from a miniature in-vacuum source mass. *Nat. Phys.* **13**, 938–942 (2017).

20. S. Restuccia, M. Toroš, G. M. Gibson, H. Ulbricht, D. Faccio, M. J. Padgett, Photon bunching in a rotating reference frame. *Phys. Rev. Lett.* **123**, 110401 (2019).

21. M. Cromb, S. Restuccia, G. M. Gibson, M. Toroš, M. J. Padgett, D. Faccio, Mechanical rotation modifies the manifestation of photon entanglement. *Phys. Rev. Res.* **5**, L022005 (2023).

22. M. Fink, F. Steinlechner, J. Handsteiner, J. P. Dowling, T. Scheidl, R. Ursin, Entanglement-enhanced optical gyroscope. *New J. Phys.* **21**, 053010 (2019).

23. G. Amelino-Camelia, Gravity-wave interferometers as quantum-gravity detectors. *Nature* **398**, 216–218 (1999).

24. A. Delgado, W. P. Schleich, G. Süssmann, Quantum gyroscopes and Gödel's universe: Entanglement opens a new testing ground for cosmology. *New J. Phys.* **4**, 37 (2002).

25. F. Bosi, G. Cella, A. Di Virgilio, A. Ortolan, A. Porzio, S. Solimeno, M. Cerdonio, J. P. Zendri, M. Allegrini, J. Belfi, N. Beverini, B. Bouhadef, G. Carelli, I. Ferrante, E. Maccioni, R. Passaquieti, F. Stefani, M. L. Ruggiero, A. Tartaglia, K. U. Schreiber, A. Gebauer, J.-P. R. Wells, Measuring gravitomagnetic effects by a multi-ring-laser gyroscope. *Phys. Rev. D* **84**, 122002 (2011).

26. Y. Margalit, O. Dobkowski, Z. Zhou, O. Amit, Y. Japha, S. Moukouri, D. Rohrlich, A. Mazumdar, S. Bose, C. Henkel, R. Folman, Realization of a complete stern-gerlach interferometer: Toward a test of quantum gravity. *Sci. Adv.* **7**, eabg2879 (2021).

27. P. Asenbaum, C. Overstreet, T. Kovachy, D. D. Brown, J. M. Hogan, M. A. Kasevich, Phase shift in an atom interferometer due to spacetime curvature across its wave function. *Phys. Rev. Lett.* **118**, 183602 (2017).

28. P. Asenbaum, C. Overstreet, M. Kim, J. Curti, M. A. Kasevich, Atom-interferometric test of the equivalence principle at the 10^{-12} level. *Phys. Rev. Lett.* **125**, 191101 (2020).

29. M. Tse, H. Yu, N. Kijbunchoo, A. Fernandez-Galiana, P. Dupej, L. Barsotti, C. D. Blair, D. D. Brown, S. E. Dwyer, A. Effler, M. Evans, P. Fritschel, V. V. Frolov, A. C. Green, G. L. Mansell, F. Matichard, N. Mavalvala, D. E. McClelland, L. McCuller, T. McRae, J. Miller, A. Mullavey, E. Oelker, I. Y. Phinney, D. Sigg, B. J. J. Slagmolen, T. Vo, R. L. Ward, C. Whittle, R. Abbott, C. Adams, R. X. Adhikari, A. Ananyeva, S. Appert, K. Arai, J. S. Areeda, Y. Asali, S. M. Aston, C. Austin, A. M. Baer, M. Ball, S. W. Ballmer, S. Banagiri, D. Barker, J. Bartlett, B. K. Berger, J. Betzwieser, D. Bhattacharjee, G. Billingsley, S. Biscans, R. M. Blair, N. Bode, P. Booker, R. Bork, A. Bramley, A. F. Brooks, A. Buikema, C. Cahillane, K. C. Cannon, X. Chen, A. A. Ciobanu, F. Clara, S. J. Cooper, K. R. Corley, S. T. Countryman, P. B. Covas, D. C. Coyne, L. E. H. Datrier, D. Davis, C. Di Fronzo, J. C. Driggers, T. Etzel, T. M. Evans, J. Feicht, P. Fulda, M. Fyffe, J. A. Giaime, K. D. Giardina, P. Godwin, E. Goetz, S. Gras, C. Gray, R. Gray, A. Gupta, E. K. Gustafson, R. Gustafson, J. Hanks, J. Hanson, T. Hardwick, R. K. Hasskew, M. C. Heintze, A. F. Helmling-Cornell, N. A. Holland, J. D. Jones, S. Kandhasamy, S. Karki, M. Kasprzack, K. Kawabe, P. J. King, J. S. Kissel, R. Kumar, M. Landry, B. B. Lane, B. Lantz, M. Laxen, Y. K. Lecoeuche, J. Leviton, J. Liu, M. Lormand, A. P. Lundgren, R. Macas,

M. MacInnis, D. M. Macleod, S. Márka, Z. Márka, D. V. Martynov, K. Mason, T. J. Massinger, R. McCarthy, S. McCormick, J. McIver, G. Mendell, K. Merfeld, E. L. Merilh, F. Meylahn, T. Mistry, R. Mittleman, G. Moreno, C. M. Mow-Lowry, S. Mozzon, T. J. N. Nelson, P. Nguyen, L. K. Nuttall, J. Oberling, R. J. Oram, B. O'Reilly, C. Osthelder, D. J. Ottaway, H. Overmier, J. R. Palamos, W. Parker, E. Payne, A. Pele, C. J. Perez, M. Pirello, H. Radkins, K. E. Ramirez, J. W. Richardson, K. Riles, N. A. Robertson, J. G. Rollins, C. L. Romel, J. H. Romie, M. P. Ross, K. Ryan, T. Sadecki, E. J. Sanchez, L. E. Sanchez, T. R. Saravanan, R. L. Savage, D. Schaetzl, R. Schnabel, R. M. S. Schofield, E. Schwartz, D. Sellers, T. J. Shaffer, J. R. Smith, S. Soni, B. Sorazu, A. P. Spencer, K. A. Strain, L. Sun, M. J. Szczepańczyk M. Thomas, P. Thomas, K. A. Thorne, K. Toland, C. I. Torrie, G. Traylor, A. L. Urban, G. Vajente, G. Valdes, D. C. Vander-Hyde, P. J. Veitch, K. Venkateswara, G. Venugopalan, A. D. Viets, C. Vorvick, M. Wade, J. Warner, B. Weaver, R. Weiss, B. Willke, C. C. Wipf, L. Xiao, H. Yamamoto, M. J. Yap, H. Yu, L. Zhang, M. E. Zucker, J. Zweizig, Quantum-enhanced advanced ligo detectors in the era of gravitational-wave astronomy. *Phys. Rev. Lett.* **123**, 231107 (2019).

30. H. Yu, L. McCuller, M. Tse, N. Kijbunchoo, L. Barsotti, N. Mavalvala, Quantum correlations between light and the kilogram-mass mirrors of LIGO. *Nature* **583**, 43–47 (2020).

31. F. Acernese, M. Agathos, L. Aiello, A. Allocca, A. Amato, S. Ansoldi, S. Antier, M. Arène, N. Arnaud, S. Ascenzi, P. Astone, F. Aubin, S. Babak, P. Bacon, F. Badaracco, M K M Bader, J. Baird, F. Baldaccini, G. Ballardin, G. Baltus, C. Barbieri, P. Barneo, F. Barone, M. Barsuglia, D. Barta, A. Basti, M. Bawaj, M. Bazzan, M. Bejger, I. Belahcene, S. Bernuzzi, D. Bersanetti, A. Bertolini, M. Bischi, M. Bitossi, M A Bizouard, F. Bobba, M. Boer, G. Bogaert, F. Bondu, R. Bonnand, B A Boom, V. Boschi, Y. Bouffanais, A. Bozzi, C. Bradaschia, M. Branchesi, M. Breschi, T. Briant, F. Brighenti, A. Brillet, J. Brooks, G. Bruno, T. Bulik, H J Bulten, D. Buskulic, G. Cagnoli, E. Calloni, M. Canepa, G. Carapella, F. Carbognani, G. Carullo, J Casanueva Diaz, C. Casentini, J. Castañeda, S. Caudill, F. Cavalier, R. Cavalieri, G. Cella, P Cerdá-Durán, E. Cesarini, O. Chaibi, E Chassande-Mottin, F. Chiadini, R. Chierici, A. Chincarini, A. Chiummo, N. Christensen, S. Chua, G. Ciani, P. Ciecielag, M. Cieślar, R. Ciolfi, F. Cipriano, A. Cirone, S. Clesse, F. Cleva, E. Coccia, P.-F. Cohadon, D. Cohen, M. Colpi, L. Conti, I Cordero-Carrión, S. Corezzi, D. Corre, S. Cortese, J.-P. Coulon, M. Croquette, J.-R. Cudell, E. Cuoco, M. Curylo, B D'Angelo, S D'Antonio, V. Dattilo, M. Davier, J. Degallaix, M De Laurentis, S. Deléglise, W Del Pozzo, R De Pietri, R De Rosa, C De Rossi, T. Dietrich, L Di Fiore, C Di Giorgio, F Di Giovanni, M Di Giovanni, T Di Girolamo, A Di Lieto, S Di Pace, I Di Palma, F Di Renzo, M. Drago,

J.-G. Ducoin, O. Durante, D D’Urso, M. Eisenmann, L. Errico, D. Estevez, V. Fafone, S. Farinon, F. Feng, E. Fenyvesi, I. Ferrante, F. Fidecaro, I. Fiori, D. Fiorucci, R. Fittipaldi, V. Fiumara, R. Flaminio, J A Font, J.-D. Fournier, S. Frasca, F. Frasconi, V. Frey, G. Fronzè, F. Garufi, G. Gemme, E. Genin, A. Gennai, A. Ghosh, B. Giacomazzo, M. Gosselin, R. Gouaty, A. Grado, M. Granata, G. Greco, G. Grignani, A. Grimaldi, S J Grimm, P. Gruning, G M Guidi, G. Guixé, Y. Guo, P. Gupta, O. Halim, T. Harder, J. Harms, A. Heidmann, H. Heitmann, P. Hello, G. Hemming, E. Hennes, T. Hinderer, D. Hofman, D. Huet, V. Hui, B. Idzkowski, A. Iess, G. Intini, J.-M. Isac, T. Jacqmin, P. Jaranowski, R J G Jonker, S. Katsanevas, F. Kéfélian, I. Khan, N. Khetan, G. Koekoek, S. Koley, A. Królak, A. Kutynia, D. Laghi, A. Lamberts, I La Rosa, A Lartaux-Vollard, C. Lazzaro, P. Leaci, N. Leroy, N. Letendre, F. Linde, M Llorens-Monteagudo, A. Longo, M. Lorenzini, V. Loriette, G. Losurdo, D. Lumaca, A. Macquet, E. Majorana, I. Maksimovic, N. Man, V. Mangano, M. Mantovani, M. Mapelli, F. Marchesoni, F. Marion, A. Marquina, S. Marsat, F. Martelli, V. Martinez, A. Masserot, S. Mastrogiovanni, E Mejuto Villa, L. Mereni, M. Merzougui, R. Metzdorff, A. Miani, C. Michel, L. Milano, A. Miller, E. Milotti, O. Minazzoli, Y. Minenkov, M. Montani, F. Morawski, B. Mours, F. Muciaccia, A. Nagar, I. Nardecchia, L. Naticchioni, J. Neilson, G. Nelemans, C. Nguyen, D. Nichols, S. Nissanke, F. Nocera, G. Oganesyan, C. Olivetto, G. Pagano, G. Pagliaroli, C. Palomba, P T H Pang, F. Pannarale, F. Paoletti, A. Paoli, D. Pascucci, A. Pasqualetti, R. Passaquieti, D. Passuello, B. Patricelli, A. Perego, M. Pegoraro, C. Périgois, A. Perreca, S. Perriès, K S Phukon, O J Piccinni, M. Pichot, M. Piendibene, F. Piergiovanni, V. Pierro, G. Pillant, L. Pinard, I M Pinto, K. Piotrzkowski, W. Plastino, R. Poggiani, P. Popolizio, E K Porter, M. Prevedelli, M. Principe, G A Prodi, M. Punturo, P. Puppo, G. Raaijmakers, N. Radulesco, P. Rapagnani, M. Razzano, T. Regimbau, L. Rei, P. Rettegno, F. Ricci, G. Riemenschneider, F. Robinet, A. Rocchi, L. Rolland, M. Romanelli, R. Romano, D. Rosińska, P. Ruggi, O S Salafia, L. Salconi, A. Samajdar, N Sanchis-Gual, E. Santos, B. Sassolas, O. Sauter, S. Sayah, D. Sentenac, V. Sequino, A. Sharma, M. Sieniawska, N. Singh, A. Singhal, V. Sipala, V. Sordini, F. Sorrentino, M. Spera, C. Stachie, D A Steer, G. Stratta, A. Sur, B L Swinkels, M. Tacca, A J Tanasijczuk, E N Tapia San Martin, S. Tiwari, M. Tonelli, A Torres-Forné, I Tosta E Melo, F. Travasso, M C Tringali, A. Trovato, K W Tsang, M. Turconi, M. Valentini, N van Bakel, M van Beuzekom, J F J van den Brand, C Van Den Broeck, L van der Schaaf, M. Vardaro, M. Vasúth, G. Vedovato, D. Verkindt, F. Vetrano, A. Viceré, J.-Y. Vinet, H. Vocca, R. Walet, M. Was, A Zadrożny, T. Zelenova, J.-P. Zendri; Virgo Collaboration; Henning Vahlbruch, M. Mehmet, H. Lück, K. Danzmann, Increasing the

astrophysical reach of the advanced virgo detector via the application of squeezed vacuum states of light. *Phys. Rev. Lett.* **123**, 231108 (2019).

32. M. Mehmet, T. Eberle, S. Steinlechner, H. Vahlbruch, R. Schnabel, Demonstration of a quantum-enhanced fiber Sagnac interferometer. *Opt. Lett.* **35**, 1665–1667 (2010).

33. J.-W. Pan, Z.-B. Chen, C.-Y. Lu, H. Weinfurter, A. Zeilinger, M. Z. Żukowski, Multiphoton entanglement and interferometry. *Rev. Mod. Phys.* **84**, 777–838 (2012).

34. M. W. Mitchell, J. S. Lundeen, A. M. Steinberg, Super-resolving phase measurements with a multiphoton entangled state. *Nature* **429**, 161–164 (2004).

35. T. Nagata, R. Okamoto, J. L. O'Brien, K. Sasaki, S. Takeuchi, Beating the standard quantum limit with four-entangled photons. *Science* **316**, 726 –729 (2007).

36. G. Bertocchi, O. Alibart, D. B. Ostrowsky, S. Tanzilli, P. Baldi, Single-photon Sagnac interferometer. *J. Phys. B: At., Mol. Opt. Phys.* **39**, 1011–1016 (2006).

37. C. Hilweg, D. Shadmany, P. Walther, N. Mavalvala, V. Sudhir, Limits and prospects for long-baseline optical fiber interferometry. *Optica* **9**, 1238 (2022).

38. G. Sagnac, L'ether lumineux demontre par l'effet du vent relatif d'ether dans un interferometre en rotation uniforme. *Comptes Rendus* **157**, 708–714 (1913).

39. E. J. Post, Sagnac effect. *Rev. Mod. Phys.* **39**, 475–493 (1967).

40. C. Greganti, P. Schiansky, I. A. Calafell, L. M. Procopio, L. A. Rozema, P. Walther, Tuning single-photon sources for telecom multi-photon experiments. *Opt. Express* **26**, 3286–3302 (2018).

41. D. R. Williams, Earth fact sheet. https://nssdc.gsfc.nasa.gov/planetary/factsheet/earthfact.html (2024).

42. H. C. Lefèvre, The fiber-optic gyroscope, a century after sagnac's experiment: The ultimate rotation-sensing technology? *C. R. Phys.* **15**, 851–858 (2014 The Sagnac effect: 100 years later/L'effet Sagnac: 100 ans après).

43. D. T. Mead, S. Mosor, paper presented at International Society for Optics and Photonics (*SPIE*, 2020), vol. 11405, p. 1140509.

44. E. de Toldi, H. Lefèvre, F. Guattari, A. Bigueur, A. Steib, D. Ponceau, C. Moluçon, E. Ducloux, J. Wassermann, U. Schreiber, paper presented at 2017 DGON Inertial Sensors and Systems (ISS), 2017.

45. S. P. Neumann, M. Selimovic, M. Bohmann, R. Ursin, Experimental entanglement generation for quantum key distribution beyond 1 gbit/s. *Quantum* **6**, 822 (2022).

46. A. J. Brady, S. Haldar, Frame dragging and the Hong-Ou-Mandel dip: Gravitational effects in multiphoton interference. *Phys. Rev. Res.* **3**, 023024 (2021).

47. T. B. Mieling, On the influence of Earth's rotation on light propagation in waveguides. *Classical Quant. Grav.* **37**, 225001 (2020).

48. M. Toroš, M. Cromb, M. Paternostro, D. Faccio, Generation of entanglement from mechanical rotation. *Phys. Rev. Lett.* **129**, 260401 (2022).

49. M. Zych, F. Costa, I. Pikovski, T. C. Ralph, Č. Brukner, General relativistic effects in quantum interference of photons. *Classical Quant. Grav.* **29**, 224010 (2012).

50. T. B. Mieling, Gupta-Bleuler quantization of optical fibers in weak gravitational fields. *Phys. Rev. A* **106**, 063511 (2022).

51. S. P. Kish, T. C. Ralph, Quantum effects in rotating reference frames. *AVS Quantum Sci.* **4**, 011401 (2022).

52. G. E. Stedman, Ring-laser tests of fundamental physics and geophysics. *Rep. Prog. Phys.* **60**, 615–688 (1997).

53. S. Moseley, N. Scaramuzza, J. D. Tasson, M. L. Trostel, Lorentz violation and Sagnac gyroscopes. *Phys. Rev. D* **100**, 064031 (2019).

54. C. Dailey, C. Bradley, D. F. Jackson Kimball, I. A. Sulai, S. Pustelny, A. Wickenbrock, A. Derevianko, Quantum sensor networks as exotic field telescopes for multi-messenger astronomy. *Astronomy* **5**, 150–158 (2021).

55. B. E. Saleh, M. C. Teich, *Fundamentals of Photonics* (John Wiley & Sons, 2019).

56. C. R. Doerr, K. Tamura, M. Shirasaki, H. A. Haus, E. P. Ippen, Orthogonal polarization fiber gyroscope with increased stability and resolution. *Appl. Optics* **33**, 8062–8068 (1994).

57. X. S. Yao, H. Xuan, X. Chen, H. Zou, X. Liu, X. Zhao, Polarimetry fiber optic gyroscope. *Opt. Express* **27**, 19984–19995 (2019).

58. R. Simon, N. Mukunda, Minimal three-component SU(2) gadget for polarization optics. *Phys. Lett. A* **143**, 165–169 (1990).
